# The Wilms’ tumor suppressor gene regulates pancreas homeostasis and repair

**DOI:** 10.1371/journal.pgen.1007971

**Published:** 2019-02-14

**Authors:** Laura Ariza, Anabel Rojas, Ramón Muñoz-Chápuli, Rita Carmona

**Affiliations:** 1 Department of Animal Biology, Faculty of Science, University of Málaga, Málaga, Spain; 2 Andalusian Center for Nanomedicine and Biotechnology (BIONAND), Málaga, Spain; 3 Instituto de Investigación Biomédica de Málaga (IBIMA), Málaga, Spain; 4 Centro Andaluz de Biología Molecular y Medicina Regenerativa-CABIMER, Universidad Pablo de Olavide, Universidad de Sevilla, Consejo Superior de Investigaciones Científicas (CSIC), Seville, Spain; 5 Centro de Investigación Biomédica en Red de Diabetes y Enfermedades Metabólicas Asociadas (CIBERDEM), Madrid, Spain; UNITED KINGDOM

## Abstract

The Wilms’ tumor suppressor gene (*Wt1*) encodes a zinc finger transcription factor that plays an essential role in the development of kidneys, gonads, spleen, adrenals and heart. Recent findings suggest that WT1 could also be playing physiological roles in adults. Systemic deletion of WT1 in mice provokes a severe deterioration of the exocrine pancreas, with mesothelial disruption, E-cadherin downregulation, disorganization of acinar architecture and accumulation of ascitic transudate. Despite this extensive damage, pancreatic stellate cells do not become activated and lose their canonical markers. We observed that pharmacological induction of pancreatitis in normal mice provokes *de novo* expression of WT1 in pancreatic stellate cells, concomitant with their activation. When pancreatitis was induced in mice after WT1 ablation, pancreatic stellate cells expressed WT1 and became activated, leading to a partial rescue of the acinar structure and the quiescent pancreatic stellate cell population after recovery from pancreatitis. We propose that WT1 modulates through the RALDH2/retinoic acid axis the restabilization of a part of the pancreatic stellate cell population and, indirectly, the repair of the pancreatic architecture, since quiescent pancreatic stellate cells are required for pancreas stability and repair. Thus, we suggest that WT1 plays novel and essential roles for the homeostasis of the adult pancreas and, through its upregulation in pancreatic stellate cells after a damage, for pancreatic regeneration. Due to the growing importance of the pancreatic stellate cells in physiological and pathophysiological conditions, these novel roles can be of translational relevance.

## Author summary

The pancreas is largely composed by an exocrine tissue organized in acini, which secrete digestive enzymes. Pancreatic stellate cells (PSC) are arranged around the acini and they can become activated by a damage and contribute to pancreas repair. The pancreas is externally covered by a mesothelium characterized by the expression of the transcription factor WT1. Loss of WT1 function in adult mice provokes a rapid and severe deterioration of the pancreas, with disorganization of the acinar tissue. Despite the extensive damage, PSC do not become activated. We first showed that a pharmacologically induced acute pancreatitis led to expression of WT1 in PSC concomitant to their activation. Then, we induced pancreatitis in mice where WT1 had been previously deleted, and the upregulation of WT1 in PSC partially rescued the repairing phenotype of the PSC and reduced the disorganization of the acinar tissue. Thus, we suggest that WT1 function is necessary to maintain the integrity of the pancreatic mesothelium and, at the same time, it is required for activation of the repairing phenotype in PSC.

## Introduction

The main cellular elements of the pancreas are basically the exocrine and the endocrine endodermal cells. However, a specific mesodermal population called the pancreatic stellate cells (PSC) is attracting the attention of many researchers due to their varied and still little-known functions in physiological and pathological conditions. PSC are usually located around the acini, ducts and blood vessels, and a subtype is also found inside the islets [1]. PSC represent about 4% of all pancreatic cells [2]. Quiescent PSC accumulate retinoids in lipid droplets and metabolize them in the same way as the hepatic stellate cells. This is relevant since pancreatic disease has been associated with impairment in pancreatic retinoid storage and metabolism [3]. PSC become activated by different stimuli and they transform into a migrating, proliferating, fibroblastoid phenotype that contributes to pancreatic fibrosis. PSC also play a central role in progression of pancreatic adenocarcinoma, contributing to the desmoplastic tissue [2,4,5].

The embryonic mesothelium expresses the Wilms’ tumor suppressor gene (*Wt1*), and this expression allows for lineage tracing of the cells delaminating from the mesothelial lining. Using this marker, it was demonstrated that a significant fraction of the liver stellate cells emerges during development from mesothelial-derived cells [6,7]. In the case of the pancreas, we have also recently described, using a transgenic murine model, that embryonic mesothelial-derived cells contribute to a major part of the PSC population, and also to a fraction of the pancreatic endothelium and the perivascular cells [8].

WT1 expression, in adult mice, is basically restricted to the mesothelium and the kidney podocytes [9]. However, when WT1 is conditionally downregulated, a lethal multiorganic failure occurs, including what was described as a massive atrophy of the pancreas [10]. Since PSC are involved in the maintenance of the pancreatic structure through the expression of β1 integrin [11] and WT1 is expressed *de novo* by activated PSC [12], we aimed to study the roles played by WT1 in both normal pancreatic mesothelium and activated PSC. Our results have shown that 1) mesothelial WT1 expression is critically required for the stability of the exocrine pancreas, and 2) WT1 expression in PSC is necessary for the control of the repairing process after a pancreatic damage. We think that these results can provide novel and interesting avenues for the knowledge and clinical management of pancreatic disease.

## Results

### WT1 expression is restricted to the pancreatic mesothelium

**Fig 1:**
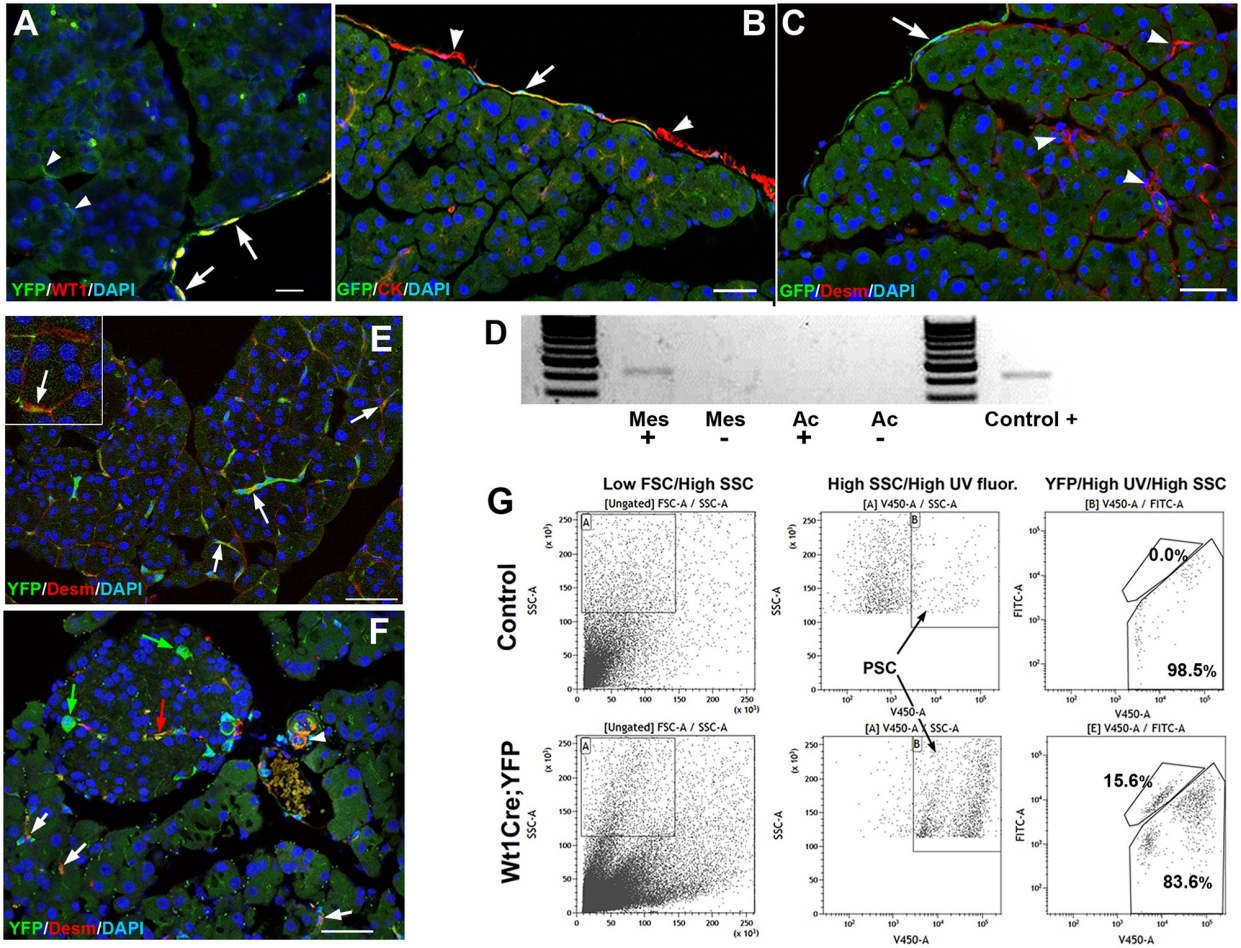
WT1 expression and *Wt1*-lineage cells in adult pancreas. **A**. *Wt1*^Cre^;R26R^EYFP^ mice. Cells derived from a *Wt1*-expressing cell lineage WT1 show YPF expression (green). Only mesothelial cells express WT1 protein (red) (arrows). Cells derived from the *Wt1*-expressing cell lineage inside the pancreas do not express WT1 (arrowheads). **B,C**. Wt1^GFP/+^ reporter mice. Expression of WT1 (green cells) is restricted to the mesothelium, although some mesothelial cells do not express the reporter WT1 (arrowheads in B). Pancreatic stellate cells (PSC), characterized by desmin expression, do not express the reporter WT1 (arrowheads in C). **D**. RT-PCR of WT1 in mesothelium (Mes) and acinar tissue (Ac) of wildtype pancreas (+) and pancreas from mice with WT1 deletion (-). Only the mesothelium from wildtype pancreas show expression of WT1. **E,F**. Part of the PSC population characterized by desmin expression also shows the *Wt1*-lineage marker YFP in both, exocrine (white arrows) and endocrine (red arrow) pancreas. Desmin+ PSC are arranged around the polygonal acini (insert in E). Note the presence of large non-PSC, *Wt1*-lineage cells inside the pancreatic islets (green arrows). The white arrowhead in F shows a desmin^+^ perivascular cell also derived from the *Wt1*-expressing lineage. **G**. FACS analysis of disaggregated pancreatic cells enriched in PSC by centrifugation on a Nikodenz solution. PSC are identified by high levels of side scatter and violet autofluorescence due to their lipidic, retinoid-containing vesicles. Comparison of the *Wt1*^Cre^;R26R^EYFP^ mice with a control mice (YFP negative) reveals that 15.6% of the PSC express the WT1-lineage marker YFP. Scale bars: A; 25 μm; B-F: 50 μm.

WT1 expression in the normal adult pancreas is restricted to the mesothelium. We have localized WT1 protein by immunohistochemistry in the mesothelial cells (Fig 1A) and we have detected only in these cells the expression of a reporter gene (WT1^GFP^ knock-in line expressing GFP under control of the *Wt1* promoter) (Fig 1B and 1C). In both cases, WT1 expression was not homogeneous, and varied between mesothelial cells.

**Fig 2:**
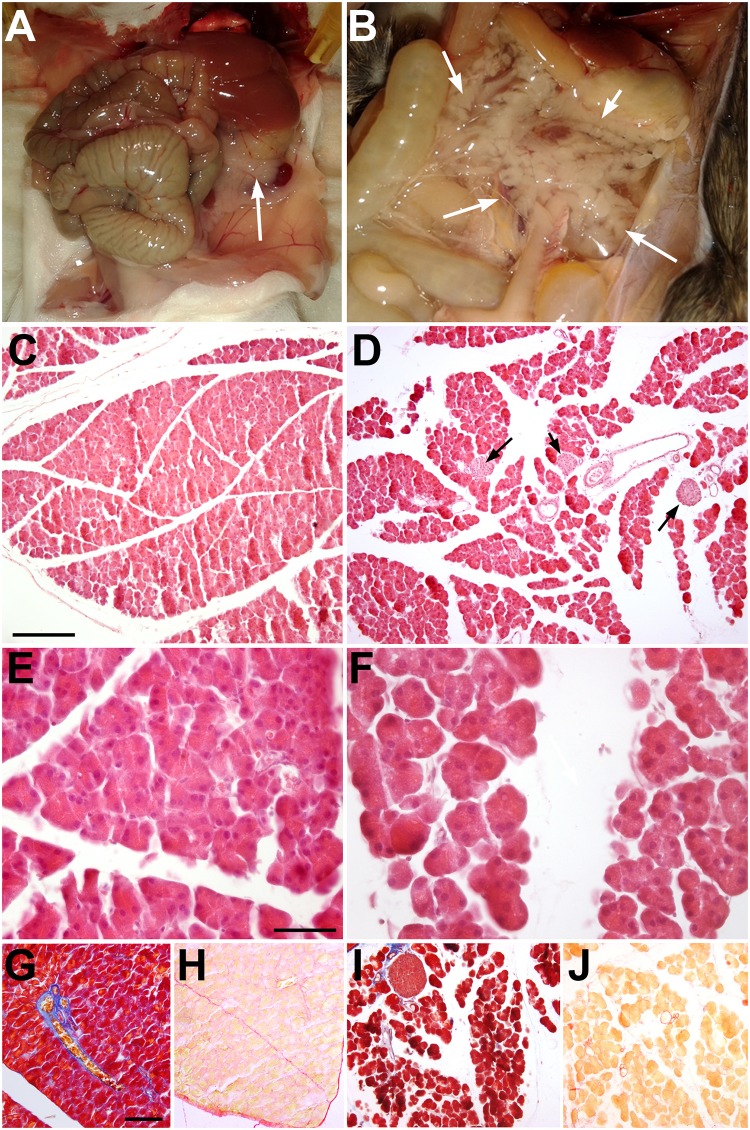
Pancreatic phenotype after systemic deletion of WT1 in the *Wt1*^CreERT2^;*Wt1*^flox^ model. **A,B**. Control (*Wt1*^CreERT2-^;*Wt1*^flox/+^) and mutant (*Wt1*^CreERT2+^;*Wt1*^flox/+^) mice injected with tamoxifen for five days and sacrificed at the ninth day after first injection. The pancreas of the mutant mice (arrows in B) is enlarged and filled with a jelly-like matrix. **C-F**. Histology of control (C,E) and mutant (D,F) pancreas, H&E staining. The acinar structure of the mutant pancreas is abnormal, showing rounded shape and reduced adhesion between cells. The islets of Langerhans appear normal in the mutant (arrows in D). **G-H**. The mutant pancreas does not show significant increase of fibrosis as shown by trichrome (I) and Sirius red staining (J). Compare with the respective controls (G and H). Scale bars: E,F: 25 μm; C,D,G-J: 100 μm.

The nature of the pancreatic mesothelium is completely different to that of other organs, since it remains loosely attached to the underlying tissue in part of its surface (Fig 2C) allowing for easy isolation by mechanical dissection. We analyzed *Wt1* expression by RT-PCR in isolated mesothelial strips and in the pancreatic tissue devoid of this layer, and we found expression only in the former, confirming the immunohistochemical data (Fig 1D).

### A part of the adult PSC population derives from WT1-expressing progenitors

A fraction of the pancreatic stroma, including part of the PSC population, derives from WT1-expressing progenitors, as shown by the *Wt1*^Cre^;R26R^EYFP^ mice, a model that allows for WT1 lineage tracing due to the constitutive YFP expression in the cells that have expressed WT1 and their descendents. The origin of PSC from WT1-expressing progenitors in the embryonic pancreas had been previously described by us [8]. A number of desmin-expressing PSC, both in the exocrine and endocrine pancreas, as well as some perivascular cells, show the WT1 lineage reporter YFP (Fig 1E and 1F). These desmin+ PSC are arranged around the polygonal acini and can be easily distinguished from the vascular muscle that it is also desmin immunoreactive. The identity of PSC was confirmed by flow cytometry analysis of disaggregated pancreas. About 15% of this population, characterized by UV-induced violet fluorescence and high side scatter [13], expressed the reporter YFP (Fig 1G).

### WT1 deletion causes deterioration of pancreas, loss of PSC markers and mesothelial disruption

The *Wt1*^CreERT2^;*Wt1*^flox^ murine line allows conditional deletion of WT1 in cells where the *Wt1* promoter is active. The pancreas of these mice treated with tamoxifen for five days developed a strong deterioration after nine days (Fig 2). However both, acinar cell size did not change significantly (Table 1). First, we confirmed the downregulation of WT1 in the mesothelium by RT-PCR (Fig 1D). After WT1 deletion, the pancreas appeared much enlarged and filled with a jelly-like, transparent matrix that increased the total pancreatic weight (Fig 2A and 2B and Table 1). Despite the increase of volume, due to the accumulation of fluid among the acini, the dry weight of the whole pancreas did not change (Table 1).

**Table 1:** Morphological and physiological features of the pancreas of mice with conditional deletion of WT1.

	Acinar cell area (μm^2^)	Fresh weight (mg)	Dry weight (mg)	CD105+ area (% DAPI area)	Amylase+ area (% DAPI area)	Amylase RNA (*2*^-ΔΔCT^)	Elastase-1 RNA (*2*^-ΔΔCT^)	PTF1A RNA (*2*^-ΔΔCT^)
Control	277,3±37,17	142,6±16,9**	40,2±3,71	11,9±2,27	268±30,9*	1,14±0,24	0,97±0,14	1,20±0,43
Mutant	234,0±9,26	492,5±78,5**	39,7±3,12	11,7±1,52	83±10,5*	0,93±0,38	0,47±0,19	0,54±0,20
N control	7	5	5	7	5	4	4	4
N mutant	9	6	6	7	5	4	4	4

Morphological and physiological features of the pancreas of mice with conditional deletion of WT1 (five days of tamoxifen, analysis at nine days). The number of histological sections or pancreas analyzed is indicated in the bottom rows. The asterisks indicate significant differences (**: p<0.01, Student’s t test; * p<0.05, U Mann-Whitney test).

Histologically, the WT1-deficient pancreas was characterized by complete disruption of the exocrine tissue architecture (Fig 2C–2F). Evidence of fibrosis was not apparent as shown by the lack of collagen accumulation (Fig 2G–2J). Islet histology was apparently not affected by the loss of WT1 expression (Fig 2D). Thus, loss of WT1 function provoked a rapid and drastic disorganization of the exocrine pancreas without loss of pancreatic mass.

**Fig 3:**
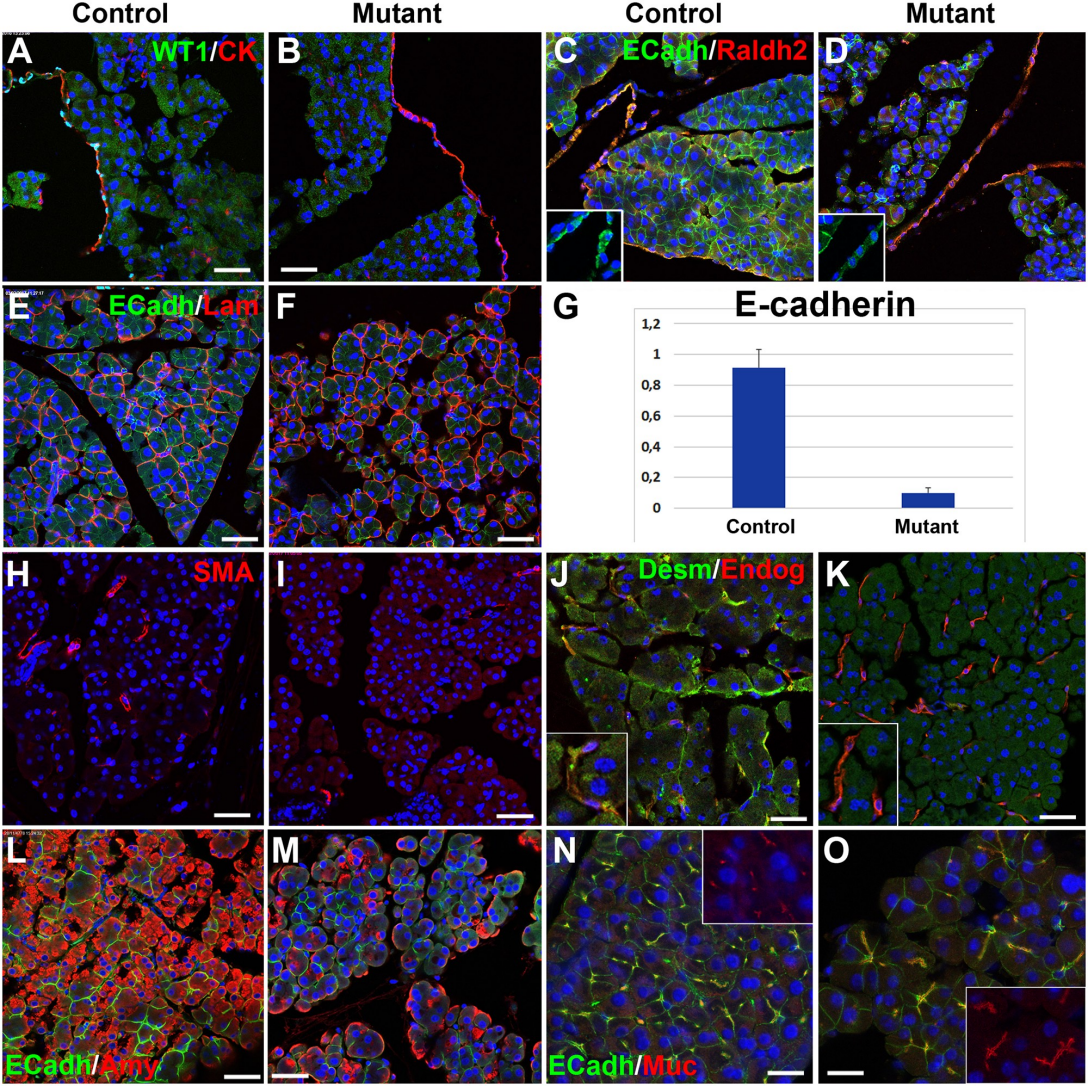
Pancreatic phenotype after systemic deletion of WT1 in the *Wt1*^CreERT2^;*Wt1*^flox^ model. **A,B**. WT1 is expressed only in the pan-cytokeratin+ mesothelial cells of control mice (A) and is deleted in the mutant mice (B). **C,D**. E-cadherin expression is downregulated in the pancreas of the mice with deletion of WT1, both in acini and mesothelium (inserts). However, mesothelial expression of RALDH2 does not change in mutant mice. **E,F**. Laminin expression is maintained in the mutant mice, but it reveals changes in the size, shape and adhesion of the acini. Note the E-cadherin immunoreactivity decrease in the mutant. **G**. E-cadherin downregulation in mutant mice is confirmed by qPCR (mean of three biological replicates, p<0.01, Student’s t test). **H,I**. Pancreatic stellate cells are not activated by the ablation of WT1, as demonstrated by the lack of SMC α-actin expression. **J,K**. Desmin^+^ pancreatic stellate cells become very scarce in the mutant mice. However, endoglin (CD105) expression is maintained in putative PSC. **L,M**. Amylase immunoreactivity is significantly reduced in mutant mice (quantified in Table 1). **N,O**. Upregulation and changes in the localization of mucin-1 immunoreactivity in mutant mice suggests loss of acinar cell polarity. Scale bars: 50 μm.

Confocal analysis confirmed the loss of WT1 in the mesothelium (Fig 3A and 3B). The retinoic acid (RA) synthesizing enzyme RALDH2 is a direct target of WT1 in the epicardium, and RA is involved in PSC stability (see discussion). Thus, we checked RALDH2 expression in the pancreas of mice after WT1 deletion. Unexpectedly, RALDH2 expression in the mesothelium appeared similar to that of controls despite the downregulation of WT1 (Fig 3C and 3D). The level of RALDH2 immunoreactivity within the pancreatic tissue was apparently reduced in these mice. This decrease was confirmed in the experiment of two days-administration of tamoxifen (see below, Fig 6E and 6N and S5 Fig). Thus, WT1 deletion provokes a decrease of RALDH2 expression in the pancreatic stroma, but not in the mesothelium.

Confocal analysis also showed a downregulation of E-cadherin in both, mesothelium and the pancreatic acini (Fig 3C–3F) in WT1-ablated mice. This drastic decrease in E-cadherin expression was also confirmed by qPCR (Fig 3G). This observation probably explains the acinar disorganization. In fact, acini, defined by laminin immunoreactivity, appeared rounded in the mutant and polygonal in controls, suggesting reduced adhesion between exocrine cells (Fig 3E and 3F).

PSC did not show upregulation of SMC α-actin expression (Fig 3H and 3I) consistently with the lack of fibrosis in WT1-ablated mice. This suggests that PSC are not activated by the pancreatic disorganization induced after conditional deletion of WT1. Furthermore, the canonical markers of PSC, desmin and GFAP, were strongly downregulated after WT1 deletion (Fig 3J and 3K and S1 Fig). The expression of Snail1, which is also characteristic of a subset of PSC [14] and is upregulated in activated PSC [15] was also strongly downregulated after the WT1 ablation (S1 Fig).

PSC are not lost by apoptosis, since we did not find upregulation of activated caspase-3 (S1 Fig). The marker of active fibroblasts, 5B5, which targets the β subunit of prolyl-4-hydroxylase, is also missing in the pancreas after WT1 ablation, consistently with the lack of fibrosis (S1 Fig). Endoglin (CD105) was normally coexpressed with desmin in PSC (S1 Fig), and these cells also expressed weakly FSP1 (S1 Fig). After WT1 ablation, endoglin expression remains in desmin-negative and FSP1-negative cells, and the frequency of these endoglin-positive cells was similar to that of desmin-positive cells in control mice (Table 1 and S1 Fig). These results suggest that PSC become dedifferentiated after WT1 ablation and remain as inactive, non-fibrogenic endoglin^+^/FSP1^-^/5B5^-^ fibroblastoid cells. We also observed, in the mutant mice, endoglin-negative cells expressing strongly FSP1 and the hematopoietic marker CD45 (S1 and S2 Figs). We will discuss below the origin and hypothetical function of these cells.

Function of acinar cells was moderately impaired by the deletion of WT1. Amylase immunoreactivity was significantly reduced (Fig 3L and 3M, quantification by image analysis in Table 1). Mucin-1 was abnormally distributed, suggesting altered polarization of the exocrine cells (Fig 3N and 3O). However, at the transcriptional level the impairment was lesser. Levels of transcripts for amylase, elastase-1 and the transcription factor PTF1A measured by qPCR were lower in mutant pancreas than in controls, although the differences were not significant at p<0,05 (Table 1).

Mice with deletion of WT1 showed an accumulation of ascitic fluid in the peritoneal cavity. No differences were found in protein concentration in this fluid between mutants and controls (0.44±0.08 vs. 0.50±0.15 g/L, N = 8 and N = 6, respectively). The low levels of proteins in the fluid indicate that the ascitic fluid is a transudate rather than an inflammatory exudate. Ascites was not related with hepatic failure, since aspartate transaminase and alanine transaminase concentration in serum were normal, and we could not find apparent damage in liver or liver mesothelium (S3 Fig). The spleen was not hypertrophied. Thus, we can discard portal hypertension as the cause of the ascites.

**Fig 4:**
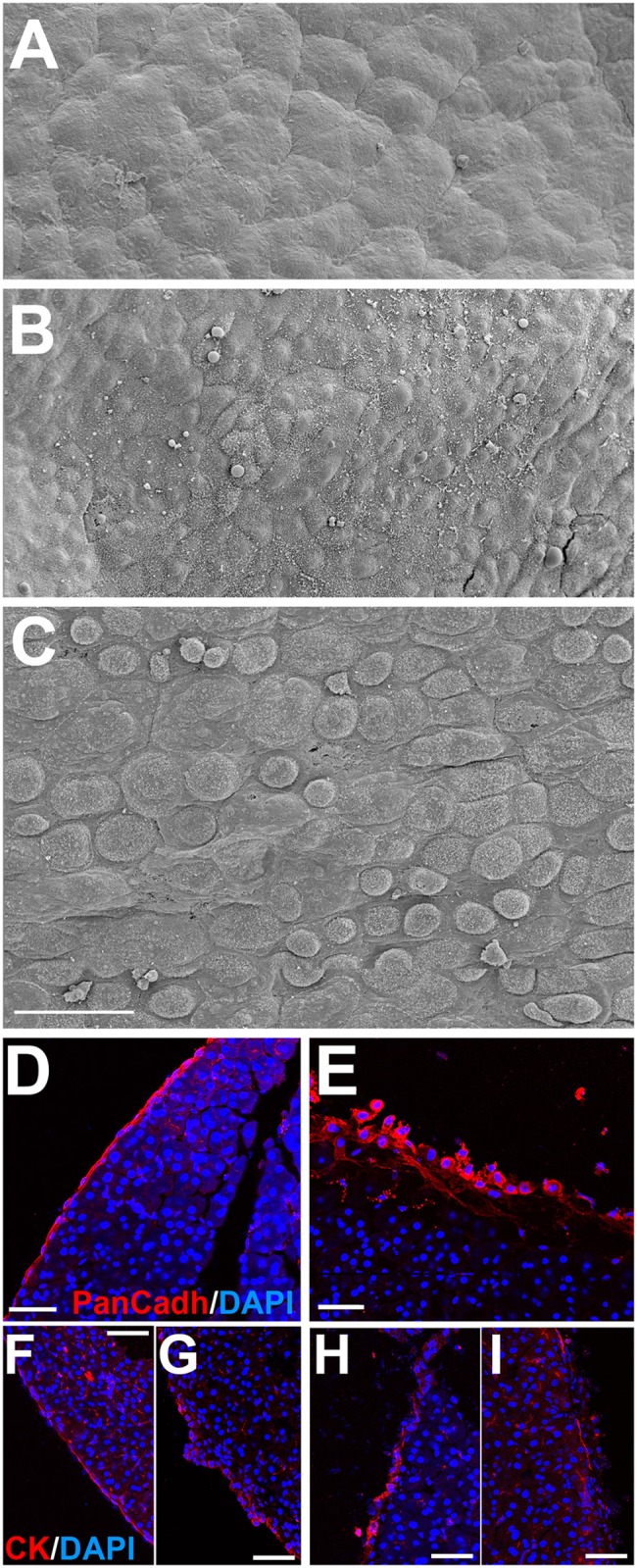
Pancreatic mesothelium is disrupted after WT1 deletion. **A**. Scanning electron micrography of normal pancreatic mesothelium. **B**. Pancreatic mesothelium of a control mice (*Wt1*^CreERT2^;*Wt1*^flox/+^) treated with tamoxifen. **C**. Pancreatic mesothelium of a mutant mice (*Wt1*^CreERT2^;*Wt1*^flox/+^) showing reduced adhesion between mesothelial cells. **D,E**. Disruption of the mesothelium of the mutant mice (E) is shown by pan-cadherin immunolocalization. **F,G**. Pan-cytokeratin immunolocalization. Different morphologies are observed in the pancreatic mesothelium of these control mice, but mesothelial cells were always forming a continuous cell layer. **H,I**. Pan-cytokeratin immunolocalization in the mutant mice reveals disruption of the mesothelium. Scale bars: 50 μm.

High levels of α-amylase in the ascitic fluid of the mutants (9.53±0.53 vs. 1.20±0.35 uKat/L in the controls, N = 8 and N = 6, p<0.001, Student’s t test) were consistent with a leakage of pancreatic enzymes into the peritoneal cavity. In fact, the pancreatic mesothelium of the mice with deletion of WT1 showed areas where intercellular junctions had disappeared and the mesothelium was disorganized, as evidenced by pan-cadherin and pan-cytokeratin immunolocalization (markers of mesothelial cells), as well as by scanning electron microscopy of the peritoneal surface (Fig 4).

### Caerulein-induced pancreatitis provokes WT1 upregulation in mesothelium as well as *de novo* expression in activated PSC

**Fig 5:**
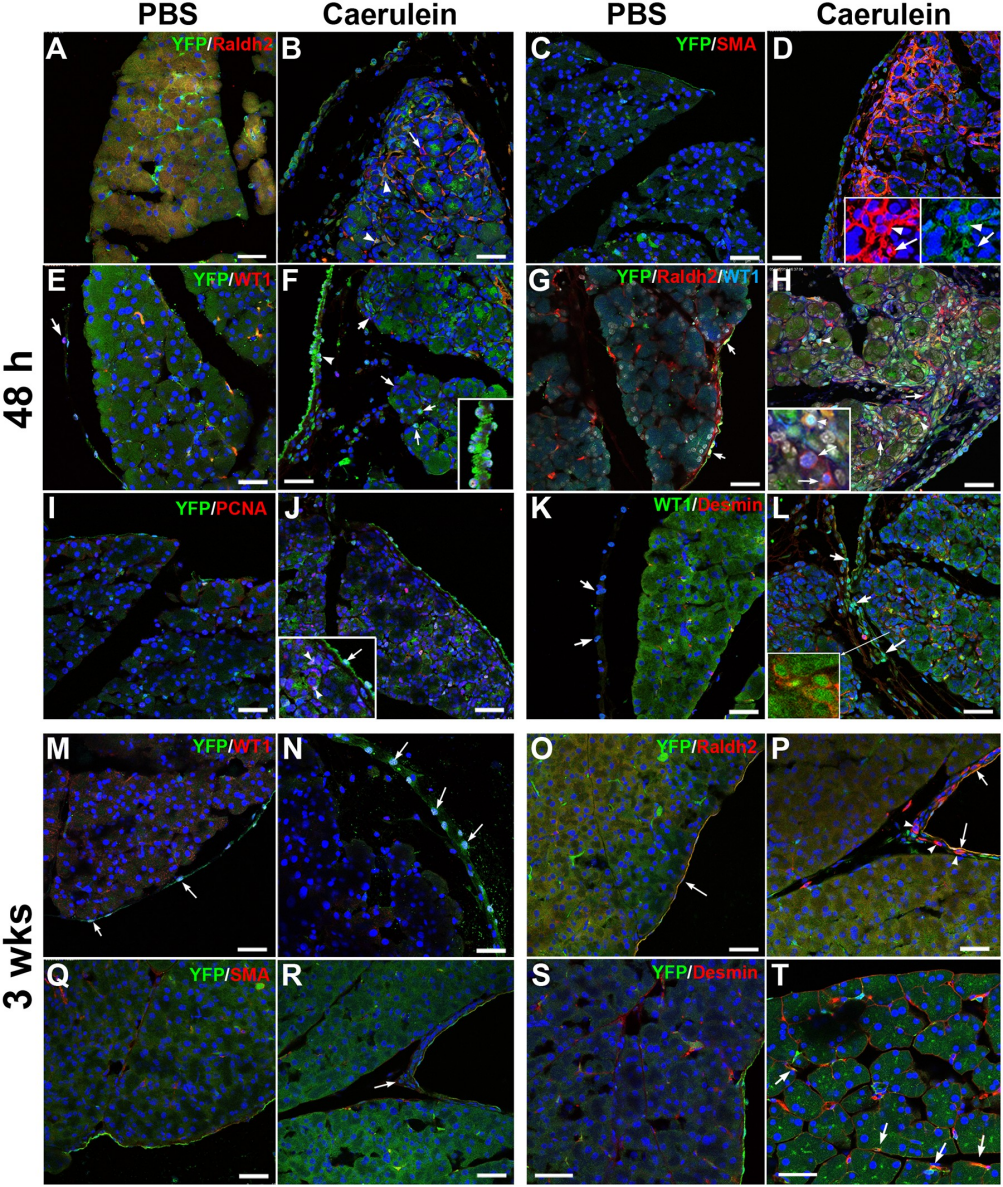
Localization of cells derived from the *Wt1*-expressing cell lineage (*Wt1*^Cre^;R26R^EYFP^ model) after induction of pancreatitis with caerulein. A-L show results obtained after 48 h of the induction and M-T show the pancreas after three weeks. **A-D**. Caerulein induced pancreatitis provokes a strong upregulation of RALDH2 and SMC α-actin in pancreatic stellate cells. Most of these activated cells belong to the *Wt1*-expressing cell lineage (arrowheads in B and D)), but some of them do not express the Wt1 lineage reporter (arrows in B and D). **E,F**. WT1 is only expressed in the mesothelium of control pancreas (arrow in E), but pancreatitis induces expression of WT1 in periacinar, putative pancreatic stellate cells (arrows in F). Note the upregulation of WT1 in the mesothelium, which is apparently releasing cells to the mesothelial space (insert). **G,H**. RALDH2 upregulation provoked by pancreatitis occurs in WT1^+^ cells, either from the *Wt1* lineage (arrowheads in H) or not (arrows in H). In the latter cells WT1 is expressed *de novo*, as demonstrated by the lack of the lineage marker YFP. In normal mice WT1 and RALDH2 expression only coincide in the mesothelium (arrows in G). **I,J**. Pancreatitis induces proliferation of Wt1-lineage cells in both, mesothelium (arrow) and periacinar areas (arrowheads). **K,L**. Desmin expression is maintained in pancreatic stellate cells after induction of pancreatitis. Wt1 expression is upregulated in the mesothelium (arrows). The insert (only red and green channels) shows desmin+ cells expressing WT1 protein. **M,N**. After three weeks of pancreatitis induction, WT1 expression is again restricted to the mesothelium (arrows). Note the accumulation of submesothelial cells in the caerulein-treated mice. **O,P**. RALDH2 expression appears in the mesothelium (arrows) but also in submesothelial cells that are not derived from a Wt1-expressing lineage (arrowheads). **Q,R**. SMC α-actin, marker of activation of pancreatic stellate cells, has disappeared after three weeks, and it is not expressed by submesothelial cells (arrow). **S,T**. After recovery of the pancreatitis, desmin+ pancreatic stellate cells are abundant and frequently express the *Wt1*-lineage marker YFP (arrows in T). Scale bars: 50 μm.

We induced pancreatitis in *Wt1*^Cre^;R26R^EYFP^ mice by injection of the secretagogue substance caerulein, an oligopeptide that acts similarly to cholecystokinin. 48 h after the caerulein treatment we observed strong activation of PSC, which expressed high levels of SMC α-actin, RALDH2, desmin (Fig 5A–5H, 5K and 5L) and proliferation markers (Fig 5I and 5J).

Activation of PSC was concomitant with a strong upregulation of WT1immunoreactivity in the pancreatic mesothelium, which showed areas with signs of delamination (Fig 5F, insert) and proliferation (Fig 5J). WT1+ cells were frequently observed in the space between the mesothelium and the pancreatic tissue (Fig 5F and 5H). Coexpression of WT1 and desmin or RALDH2 indicates that PSC express WT1 during their activation (Fig 5H and 5L, inserts). Interestingly, some of the WT1+/RALDH2+, putative PSC were YFP-, i.e., they were expressing WT1 *de novo*, before activation of the lineage reporter (Fig 5H, insert).

As expected, three weeks after caerulein treatment of the Wt1^Cre^;R26R^EYFP^ mice, the pancreas was fully recovered from the induced pancreatitis and showed normal expression levels of WT1, RALDH2 and PSC markers. A closer view of the recovered pancreas after caerulein treatment shows accumulation of cells below the mesothelium (Fig 5M–5R). The lack of the YFP reporter expression suggested that the submesothelial cells were not derived from WT1-expressing cells (Fig 5P and 5R). WT1 was still strongly expressed in many mesothelial cells, but it was downregulated in the PSC (Fig 5M and 5N) together with the markers of activation, RALDH2 and SMCα-actin (Fig 5O–5R). However, PSC were expressing normally desmin, frequently in colocalization with the WT1 lineage marker YFP (Fig 5S and 5T). Thus, most quiescent PSC derive from WT1-expressing cells after pancreatitis.

### Caerulein-induced pancreatitis partially rescues the PSC population and the acinar organization in WT1-ablated mice

Since we have observed that caerulein-induced pancreatitis provoked *de novo* WT1 expression in the pancreatic stroma, and particularly in PSC, we checked the effect of this treatment in mice with conditional deletion of WT1 that normally would develop a severe pancreatic disorganization. We injected *Wt1*^CreERT2^;*Wt1*^flox^ control (Wt1^CreERT2-^;Wt1^flox/+^) and mutant (Wt1^CreERT2+^;Wt1^flox/+^) mice with tamoxifen (two days) and after 48 h we treated the mice with caerulein or PBS. Previously, we confirmed that WT1 deletion *per se* did not involve activation of PSC or *de novo* expression of WT1 in these cells (S4 Fig).

**Fig 6:**
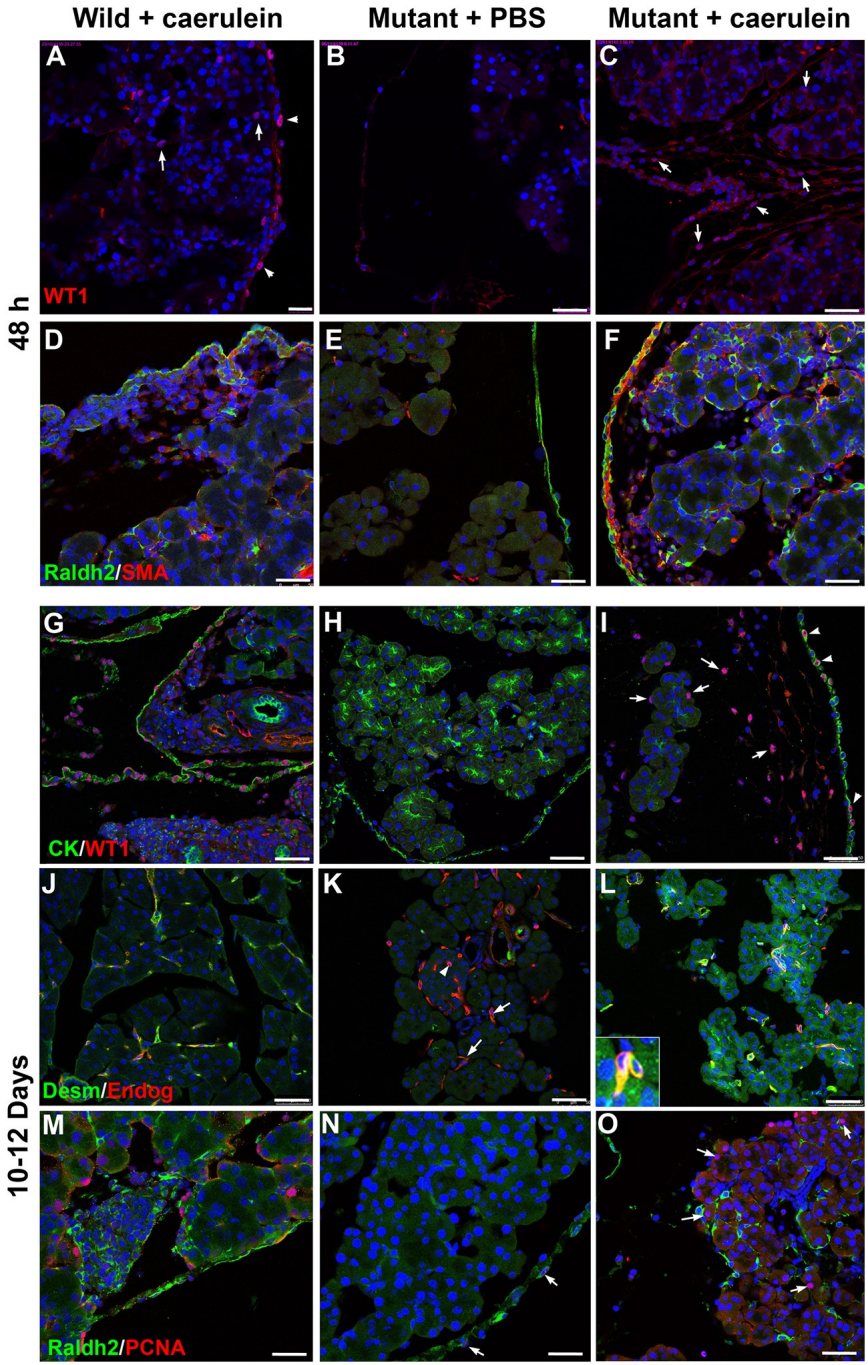
Pancreatic phenotype after systemic deletion of WT1 in the *Wt1*^CreERT2^;*Wt1*^flox^ model and induction of pancreatitis with caerulein. A-F show results obtained after 48 h and G-O represent the pancreas after 10–12 days, when mice have recovered from pancreatitis. **A-C**. WT1 is expressed in mesothelial and stromal cells of the mouse with pancreatitis (A), it is lacking in mice after deletion of WT1 (B), but it is expressed in stromal cells after induction of pancreatitis (arrows in C). **D-F**. Markers of pancreatic stellate cell activation (SMC α-actin, RALDH2) are upregulated by induction of pancreatitis, even when WT1 expression has been ablated as shown in F. **G-I**. WT1 expression persists in mice with WT1 deletion after recovery of pancreatitis (arrows in I), and this expression appears even in some pan-cytokeratin+, mesothelial cells (arrowheads in I). **J-L**. The number of desmin+ expressing cells (putative stellate cells) increase in the pancreas with WT1 deletion after pancreatitis (arrows in L). These cells lose the desmin immunoreactivity in WT1-deficient mice, but they keep the CD105 expression (arrows in K) even in the islets (arrowhead). **M-O**. Proliferating cells are abundant in the pancreatic stroma of mice with WT1 deletion after recovery of pancreatitis (arrows in O), but they are very scarce and limited to the mesothelium in mutant mice that have not suffered of pancreatitis (arrows in N). Note the increase in RALDH2 in the mutant mice after recovery of pancreatitis (quantified in S5 Fig). Scale bars: A = 50 μm, B-O = 50 μm.

After 48 h, control mice treated with caerulein showed upregulation of WT1 in both mesothelium and stromal cells (Fig 6A), while no WT1 expression was observed in mutant mice injected with PBS (Fig 6B) confirming efficient excision of the WT1 floxed allele. Caerulein-treated mutant mice did not display WT1 expression in the mesothelium (Fig 6C) but a remarkable number of WT1-expressing cells were observed in the submesothelial space and inside the pancreatic parenchyma (Fig 6C). As expected, PSC appeared activated in caerulein-treated control mice, expressing SMC-α actin and RALDH2 (Fig 6D). Despite the inactivation of WT1 in the mesothelium of *Wt1*^CreERT2^;*Wt1*^flox^mice, PSCs activation was also observed in these mice when treated with caerulein but not with PBS (Fig 6E and 6F). Thus, caerulein-induced pancreatitis activates PSC even when WT1 has been previously deleted from the mesothelium.

After 12 days of caerulein treatment, differences in acinar cells and PSC markers between mutant mice treated with caerulein or PBS were remarkable. The area and perimeter of the acini was partial, but significantly, recovered (S5 Fig). In caerulein-injected mutant mice, many WT1-expressing cells were found between the mesothelium and the pancreatic tissue, and also surrounding the acini (Fig 6G–6I). FSP1+/CD45+ cells were more frequent in the caerulein-injected mutant mice (S2 Fig). In these mice a population of desmin-expressing, endoglin+, putative quiescent PSC, was observed around the acini, suggesting that part of the PSC population had been rescued (Fig 6J–6L). Quantification of this population by image analysis revealed a two-fold increase of the surface covered by desmin immunoreactive cells (S5 Fig). The mesothelium, submesothelial cells and many stromal cells expressed RALDH2 (Fig 6M–6O). The increase of RALDH2 immunoreactivity, quantified by image analysis, was significant (S5 Fig). In order to check if this upregulation of RALDH2 provoked an increase in the accumulation of retinoids by the PSC, we analyzed by flow cytometry the fraction of low-density pancreatic cells isolated by centrifugation over Nykodenz. The violet autofluorescence induced by UV light of these cells also showed a recovery of the PSC from caerulein-treated mutant mice (S5 Fig), although the number of mice analyzed (N = 3 by group) did not allow statistical analysis.

Finally, proliferation markers showed also a higher expression in the caerulein-treated mice (Fig 6M–6O). In summary, all the features of the mutant mice injected with caerulein suggested a partial rescue of the severe phenotype provoked by the deletion of WT1.

## Discussion

In adults, the *Wt1* gene is expressed mainly in the kidney podocytes, mesothelial cells and a minor fraction of bone marrow cells [9]. Thus, it was surprising the severe phenotype of the adult deletion of WT1 in mice, characterized by a multiorgan failure and the death of the animal in a few days [10]. One of the features of this phenotype was described as a severe pancreatic atrophy. This was unexpected, since WT1 function had not previously been related with the physiology of the adult pancreas. We aimed to know better what the precise roles played by WT1 in the pancreas could be as well as the mechanisms leading to such a rapid pancreatic deterioration. Since the adult deletion of WT1 is systemic, we cannot formally discard that anomalies in the function of other organs can influence in the pancreatic phenotype. However, we think that our findings support a robust model on a novel and significant role played by WT1 for pancreas homeostasis and regeneration after damage.

First of all, the normal pancreatic expression of WT1 in adults is clearly restricted to the mesothelium. This pancreatic mesothelium seems to be different to that covering other organs, since it is loosely attached to the pancreatic parenchyma, and it shows morphological variation ranging from squamous to cuboidal. Although WT1 is not normally expressed in the pancreatic stroma, a substantial part of these cells, including many PSC, are derived from a WT1-expressing cell lineage as shown by the WT1-lineage marker YFP. This specific subpopulation of adult PSC probably originates during embryonic development from the mesothelium, since we have previously shown that more than 50% of the stromal cells derive from a WT1-expressing cell lineage, and there is no substantial postnatal generation of PSC from the mesothelium in normal conditions [8].

Loss of WT1 expression in mesothelial cells causes a rapid disorganization of the acinar architecture and also of areas of the mesothelium, leading to leakage of pancreatic secretion into the peritoneal cavity. This observation is consistent with the strong downregulation of E-cadherin expression observed in both, mesothelium and acini, after WT1 deletion. Amylase content in acinar cell decreases, and the abnormal localization of mucin-1 reveals altered polarization of acinar cells. The pancreas becomes swollen with a hyaline matrix, probably due to degradation of the normal extracellular matrix. Pancreatic weight increases threefold, although its dry weight does not change, indicating the presence of a severe edema. Surprisingly, this process does not involve the activation of pancreatic stellate cells which, instead, lose their differentiation markers, and do not express the activation marker smooth muscle α-actin nor the fibroblastic markers 5B5 and FSP1. In fact, the only FSP1-expressing cells found in the WT1-deficient pancreas are CD45+. These markers are found in the so-called “fibrocytes”, recruited from circulation in chronic inflammatory processes [16]. PSC probably dedifferentiate after WT1 ablation into inactive, non-fibrogenic fibroblastoid cells that can still be identified by the expression of the TGFβ accessory receptor endoglin (CD105). Thus, the deterioration of the pancreas provoked by WT1 deletion does not involve a noteworthy fibrosis. The significance of this finding will be discussed below.

Pancreatic disorganization and mesothelial degeneration are probably related with the ascites observed in mice with deletion of WT1. This condition was not caused by inflammation, liver failure or portal hypertension, since protein concentration in the ascitic fluid was low, serum transaminases were normal and the spleen was not hypertrophied. Thus, leakage of interstitial fluid into the coelomic cavity, probably due to the mesothelial damage (as shown in Fig 4), is the most probable cause of the ascites.

Our observations can be explained in two ways. First, it is possible that the pancreatic mesothelium secretes some WT1-dependent signal(s) required for the stability of the pancreas structure. Retinoic acid signaling might be a good candidate, due to the close relationships between WT1 and this pathway [17], and also due to the role of PSC as retinoid store [3]. However, we have not detected a downregulation of RALDH2 in the mutant mesothelium. A second and more likely explanation could be related with the critical role played by the mesothelium in providing a physical barrier to contain morphogenic signals during development [18]. This role might be conserved in the adult pancreas, where the mesothelial integrity can be required for maintaining a suitable extracellular milieu necessary for pancreatic stability. Thus, we can conclude that the WT1-dependent integrity of the pancreatic mesothelium is critical for the homeostasis of the exocrine pancreas, because this tissue works as a source of molecular signals and/or an essential physical barrier.

In any case, the disorganization of the pancreatic cytoarchitecture must be related with the stabilizing function of the PSC. Loss of β1 integrin expression in PSC leads to a pancreatic exocrine dysfunction and disturbed acinar cell–cell contacts, a phenotype similar to that herein described [11]. It has been also described that deletion of Snail1 in pancreatic mesenchymal cells (most probably a subpopulation of PSC) leads to loss of acinar structures and their replacement by adipose tissue [14]. This phenotype might be related with the observed by us after WT1 deletion, although the latter phenotype develops faster, and it involves pancreatic edema and ascites.

When we provoked a pancreatitis in the *Wt1*-lineage tracing model *Wt1*^Cre^;R26R^EYFP^, WT1 was strongly expressed not only in the mesothelium but also in PSC. This observation had been already reported [12]. PSC also expressed markers of activation and proliferation, and they strongly upregulated RALDH2. Our model of *Wt1*-lineage tracing showed that WT1 was expressed in cells of this lineage, and also *de novo* in other PSC, but always associated with the RALDH2 upregulation. Markers of PSC, such as desmin, were not lost during activation, at least in a part of the PSC population.

Thus, pancreatic damage induces an activation of WT1 in PSC, and this could be related with the upregulation of its transcriptional target RALDH2. Importantly, RA-signaling in PSC seems to be critically involved in their stabilization and quiescence [19–22]. WT1 could be modulating through the RALDH2/RA axis the restabilization of a part of the PSC population, and, indirectly, the regeneration of the pancreatic architecture after the damage, since quiescent PSC are required for exocrine pancreas stability [11] and regeneration [23]. Reversion of activated stellate cells to the quiescent phenotype has also been described in the liver [24]. We think that this hypothesis explains well the pancreatic phenotype observed in the model of WT1 deletion where damage is initially produced by the mesothelial degeneration. When WT1 expression is prevented in PSC by reiterated tamoxifen injection (5 days), activation of PSC mediated by WT1 is inhibited, the WT1/RALDH2/RA signaling axis is blocked, the PSC population dedifferentiates and the pancreas cannot recover its normal organization, which depends on the function of quiescent PSC.

In order to test this hypothesis, we tried to rescue the pancreatic phenotype of the mutant through an induction of pancreatitis. We reasoned that this induction should activate a *de novo* WT1 expression in the PSC after a short term tamoxifen-induced WT1 deletion. We previously confirmed that injection of tamoxifen for two days ablated WT1 expression and did not induce PSC activation. Our results clearly showed that PSC were activated by the caerulein treatment of the mice with WT1 deletion, and WT1/RALDH2 expression was increased in both stromal and submesothelial cells. Recruitment of FSP1+/CD45+ cells was also improved in mutant mice after pancreatitis induction.

A normal population of desmin-expressing, quiescent PSC was found in the pancreas of these mutant mice treated with caerulein 12 days after the treatment, but the mutant mice not treated with caerulein lacked of these cells. The caerulein-treated mutant mice also showed larger size of the acini. Thus, the induced pancreatitis in mice with deletion of WT1 and the subsequent *de novo* expression of WT1 in PSC partially rescued the acinar organization and, importantly, a portion of the quiescent PSC population after recovery from pancreatitis. However, normal pancreatic function was not fully rescued, and this emphasizes that mesothelial expression of WT1 is indispensable for complete regeneration of the pancreas after damage.

We think that our findings explain the pancreatic phenotype resulting of the adult WT1 deletion and they also provide new and relevant knowledge about the critical role played by WT1 in the homeostasis and repair of the pancreas. Due to the growing importance of the PSC function in physiological and pathophysiological conditions, we are aware that this novel knowledge can be of translational relevance.

## Materials and methods

### Ethics statement

The animals used in our research program were handled in compliance with the institutional and European Union guidelines for animal care and welfare. The procedures used in this study were approved by the Committee on the Ethics of Animal Experiments of the University of Malaga (procedure code 17-2018-A).

The WT1^GFP^ knockin line [25] in which the exon 1 of a WT1 allele has been replaced by the GFP sequence, was used as a reporter for active WT1 transcription. The Tg(WT1-cre)#Jbeb (WT1^cre^) mouse line has been used in previous studies to trace the WT1-expressing cell lineage or to delete specific genes in WT1-expressing cells [26–30]. For lineage tracing studies, homozygote WT1^Cre^ were crossed with homozygote Rosa26^EYFP^ (B6.129X1-Gt(ROSA)26Sortm1(EYFP)Cos/J) mice to generate permanent reporter expression in the lineage of WT1-expressing cells.

The WT1tm2(cre/ERT2)Wtp/J knockin line allows for inducible Cre-recombinase expression in WT1-expressing cells after tamoxifen treatment. We have used this line crossed with Rosa26^EYFP^ for inducible reporter expression in the WT1-expressing cell lineage [31]. Wt1tm2(cre/ERT2)Wtp/J mice were used also to delete expression of WT1 when the line was crossed with homozygote Wt1^LoxP^ mice. Cre recombinase was induced in these mice by gavage administration of tamoxifen (Sigma, T5648) (0.1 mg/g body weight dissolved in corn oil) for 2 or 5 days depending on the experiment [10]. Animals were returned to standard housing conditions prior to being killed by cervical dislocation.

### Immunocytochemistry and image analysis

After sacrifice of the mice, whole pancreas was excised, washed in PBS, fixed overnight in 2% fresh paraformaldehyde solution in PBS and frozen or paraffin embedded. Confocal immunofluorescence was performed using routine protocols. Deparaffinized sections or cryosections were rehydrated in Tris-PBS (TPBS) and blocked for non-specific binding with SBT (16% sheep serum, 1% bovine albumin, 0.1% Triton X-100 in TPBS).. Single immunofluorescence was performed incubating the sections with the primary antibody overnight at 4°C, washing in TPBS and incubating with the corresponding fluorochrome-conjugated secondary antibody. Double or triple immunofluorescence was performed by mixing both primary antibodies (chicken polyclonal, rabbit polyclonal and mouse or rat monoclonal), and incubating overnight at 4°C. In the case of the double endoglin/desmin immunostaining we incubated overnight the sections with the anti-CD105 antibody, we then blocked the sections with monovalent donkey anti-mouse IgG, and we incubated the sections again with the desmin antibody, before incubation with fluorochrome-conjugated secondary antibodies. Nuclei were counterstained with DAPI (Sigma D4592). Details of the antibodies used in this study are provided in S1 Table. Micrographs were obtained in a Leica TCS SP8 confocal microscope. The micrographs used for the figures were representative of a minimum of three biological replicates.

Image analysis was performed on confocal images obtained always under the same conditions, using the ImageJ 1.44p software and a macro tool developed by us. RGB channels were split, a threshold was applied to all the images, and the area of the cells labeled with the antibody was expressed as percentage of the DAPI+ area.

### Flow cytometry

For flow cytometry analysis, 1–2 mm^3^ fragments of pancreas were excised, dissociated for 15 min at 37°C in 0.1% collagenase solution in PBS and homogenized by repeated pipetting. The cell suspension was washed in PBS plus 2% fetal bovine serum and 10 mM HEPES. Enrichment of the PSC fraction was performed by centrifugation of the cell suspension (20 min, 1400 g) over a 13% (w/V) solution of Nykodenz in PBS [13]. The lower density of the PSC allows isolation of an enriched cell fraction that was analyzed in a FACS Verse cytometer. We used a gating strategy for cells with high levels of side scatter and violet autofluorescence. The violet autofluorescence of PSC is due to the presence of intracellular retinoids. This strategy has been previously described for isolation of hepatic stellate cells [13].

### Semiquantitative and qPCR

Total RNA was extracted from adult pancreas using NucleoSpin RNA XS (Macherey Nagel) according to the manufacturer’s instructions and quantified by a Nanodrop ND-1000 Spectrophotometer. For semiquantitative PCR, 1μg of RNA were used to synthesize cDNA with the First Strand Amplification Kit (Roche), and PCR was performed using primers described in S2 Table. The following PCR program was used for amplification: 95°C for 5 mins; 95°C for 45 sec; 60°C for 45 sec; 72°C for 45 sec; 72°C for 5 min; 30–36 cycles. For qRT-PCR analyses, RNA (1μg) was reverse transcribed with iScript Kit (Bio-Rad). Real time PCR experiments were performed with 1 μL of cDNA, SsoFast EvaGreen-mix (Bio-Rad) and corresponding primer sets. All qPCRs were performed using a LightCycler96 thermocycler (Roche) following the manufacturer’s recommendations. The relative level of expression of each gene was calculated by using Gapdh and β-actin as internal control [32]. Each PCR reaction was carried out in triplicate and repeated in at least three distinct pooled biological samples to obtain representative means.

### Ascitic fluid analysis

Ascites is the abnormal accumulation of fluid within the peritoneal cavity. Samples of ascitic fluid were analyzed in order to determine protein concentration and α-amylase activity. Protein content was analyzed through the Biuret method and α-amylase activity was measured kinetically at 37°C using the CNPG3 substrate (Spinreact).

### Induction of acute pancreatitis

Pancreatitis was induced in mice by intraperitoneal injection of 50 μg/kg of the secretagogue caerulein (Sigma C9026) in PBS buffer. Caerulein was administered as 8 daytime doses, 1 hour apart over 2 consecutive days [33,34]. Control mice received equal volumes of PBS buffer injected intraperitoneally following the same pattern.

### Statistics

All the quantitative data are expressed as mean±SEM. Data were analyzed by non parametric statistics (U Mann-Whitney test) unless indicated otherwise.

All relevant data are available in the Supporting Information File (S1 File).

## Supporting information

S1 Fig Additional information on the pancreatic phenotype after systemic deletion of WT1 in the *Wt1*^CreERT2^;*Wt1*^flox^ model. The pancreatic stellate cell marker GFAP (A,B), the apoptosis marker activated caspase-3 (D,E), and the fibroblastic marker proline-hydroxilase (5B5) (F,G) are all downregulated in pancreas with deletion of WT1. Snail1 expression is also strongly downregulated in pancreas after WT1 ablation (C, mean of three biological replicates, p<0.001, Student’t t test). However, endoglin (CD105) shows no changes (H-K), although the weak expression of FSP1 in the pancreatic stellate cells disappears (J,K, arrowheads in the inserts). Note the presence of CD105-negative cells expressing high levels of FSP1 (arrows in K). Scale bars: B,D = 25 μm, other figures = 50 μm.
(TIF)

S2 Fig Recruitment of circulating FSP1+/CD45+ cells into the pancreas after WT1 deletion. **A,B**. A few positive cells appear in the pancreas nine days after the WT1 deletion (arrows in B). The weak FSP1 immunoreactivity of the stellate cells (arrowheads in A) has disappeared. A CD45+ cell is observed in the control pancreas (arrow in A). There is not inflammatory infiltrate in the WT1-deficient pancreas. **C-F**. The FSP1+/CD45+ cell recruitment is higher in the pancreas after recovery of the induced pancreatitis in both, control (C,D) and WT1-deficient mice (E,F). Scale bar (for all the panel): 25 μm.
(TIF)

S3 Fig Liver phenotype after systemic deletion of WT1 in the *Wt1*^CreERT2^;*Wt1*^flox^ model. **A**: control. **B**. mutant. The mesothelium shows no changes after ablation of WT1. Scale bars: 50 μm.
(TIF)

S4 Fig Deletion of WT1 in the *Wt1*^CreERT2^;*Wt1*^flox^ model. Two doses of tamoxifen are enough for WT1 ablation in the pancreatic mesothelium after five days (arrows in A,B), but this treatment did not activate pancreatic stellate cells, as demonstrated by the lack of upregulation of RALDH2 and SMC α-actin (C,D). Scale bars: 50 μm.
(TIF)

S5 Fig Image analysis of histological sections in control pancreas, caerulein-induced pancreatitis, deletion of WT1 and deletion of WT1 plus induction of pancreatitis. The number of sections analyzed (always obtained from >2 mice per group) is indicated on the figure. The same sections were analyzed for acinar area and perimeter. The percentage of violet-fluorescent cells isolated by centrifugation on Nykodenz solution is also shown. Violet autofluorescence indicates accumulation of retinoids in pancreatic stellate cells. Mean acinar perimeter, mean acinar area, percentage of RALDH2+ and desmin^+^ area relative to DAPI^+^ area (nuclei) showed significantly higher values in mice with WT1 deletion after recovery of pancreatitis compared with mice with WT1 deletion (U-Mann Whitney test, p<0.05). The decrease of RALDH2 and desmin immunoreactivity was also significant when comparing control and WT1-deficient mice. Statistical comparison was not applied to the percentages of violet-fluorescent cells because of the low number of biological replicates (N = 3 per group), but the results show the same tendency as the other ones.
(TIF)

S1 Table Antibodies used in this study. (PDF)

S2 Table Primers used in this study. (PDF)

S1 File Supporting information file. (XLSX)
