# Supplementary figures and images for: The Wilms’ tumor suppressor gene regulates pancreas homeostasis and repair
Source: PLoS Genet. 2019 Feb 14;15(2):e1007971. doi: 10.1371/journal.pgen.1007971 (PMC6392337; doi:10.1371/journal.pgen.1007971)

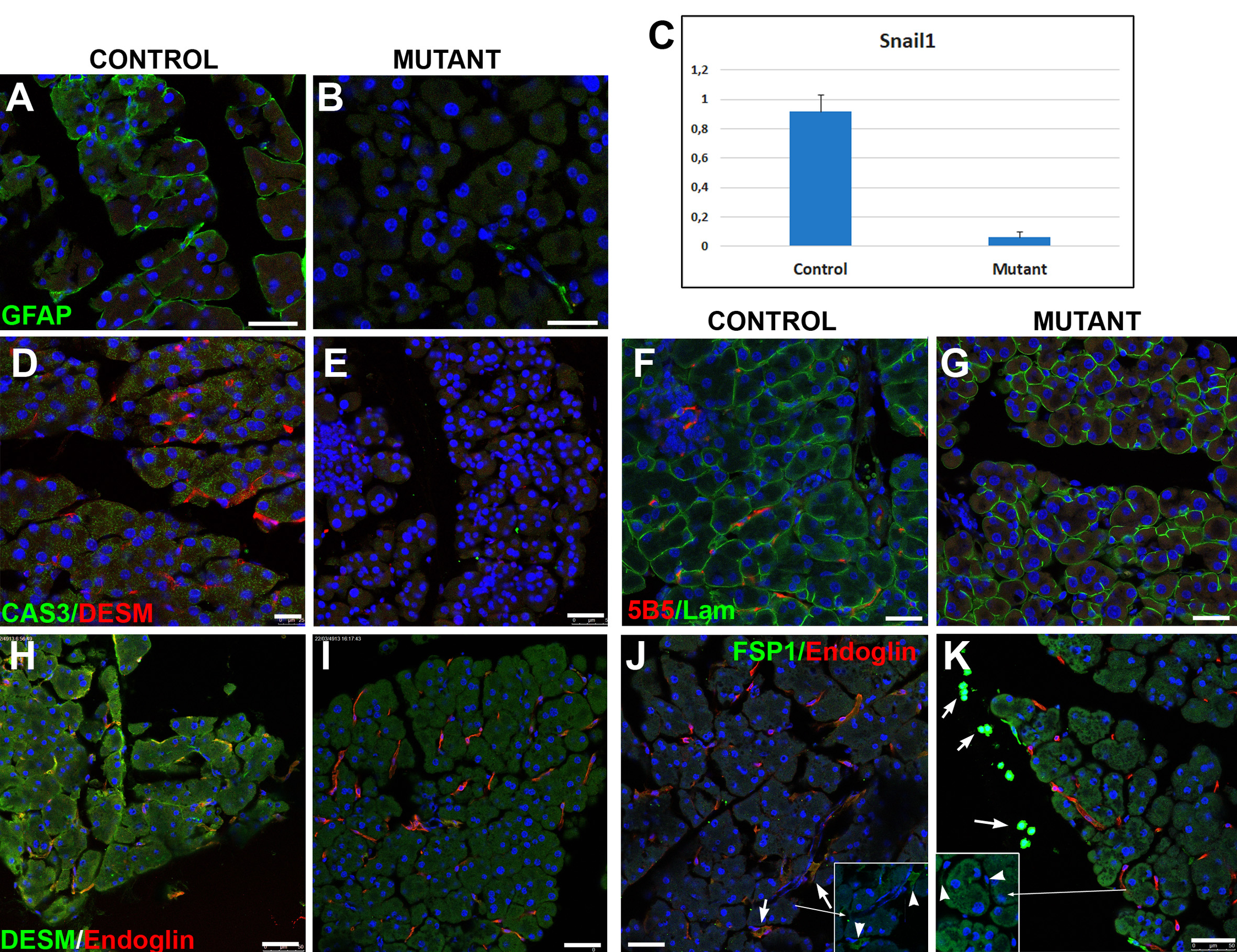

Supplement: S1 Fig — The pancreatic stellate cell marker GFAP (A,B), the apoptosis marker activated caspase-3 (D,E), and the fibroblastic marker proline-hydroxilase (5B5) (F,G) are all downregulated in pancreas with deletion of WT1. Snail1 expression is also strongly downregulated in pancreas after WT1 ablation (C, mean of three biological replicates, p<0.001, Student’t t test). However, endoglin (CD105) shows no changes (H-K), although the weak expression of FSP1 in the pancreatic stellate cells disappears (J,K, arrowheads in the inserts). Note the presence of CD105-negative cells expressing high levels of FSP1 (arrows in K). Scale bars: B,D = 25 μm, other figures = 50 μm. (TIF) [file pgen.1007971.s001.tif]

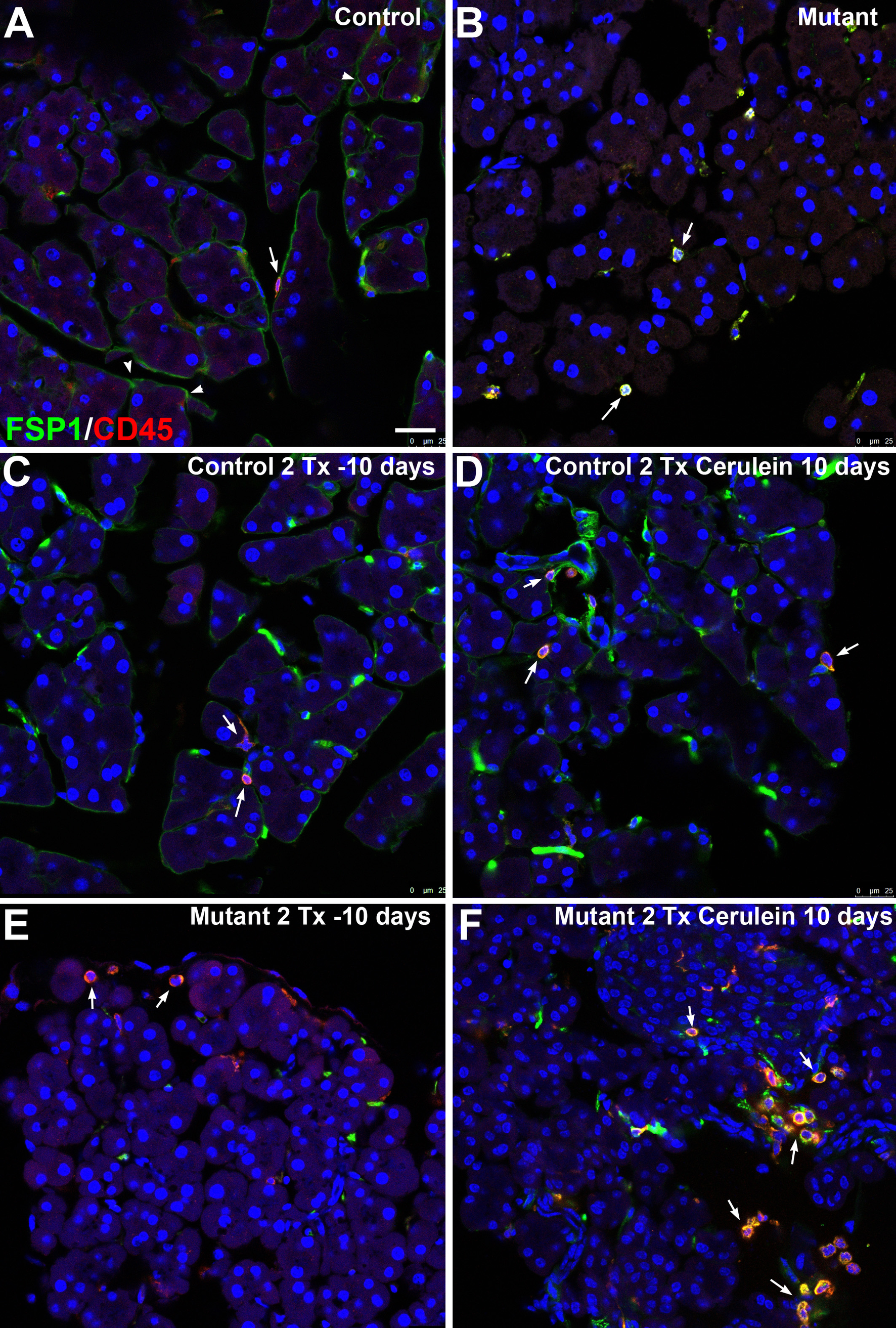

Supplement: S2 Fig — A,B. A few positive cells appear in the pancreas nine days after the WT1 deletion (arrows in B). The weak FSP1 immunoreactivity of the stellate cells (arrowheads in A) has disappeared. A CD45+ cell is observed in the control pancreas (arrow in A). There is not inflammatory infiltrate in the WT1-deficient pancreas. C-F. The FSP1+/CD45+ cell recruitment is higher in the pancreas after recovery of the induced pancreatitis in both, control (C,D) and WT1-deficient mice (E,F). Scale bar (for all the panel): 25 μm. (TIF) [file pgen.1007971.s002.tif]

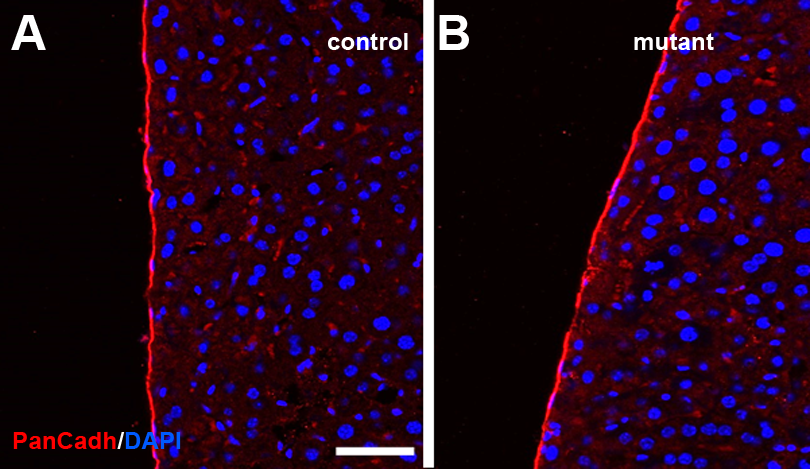

Supplement: S3 Fig — A: control. B. mutant. The mesothelium shows no changes after ablation of WT1. Scale bars: 50 μm. (TIF) [file pgen.1007971.s003.tif]

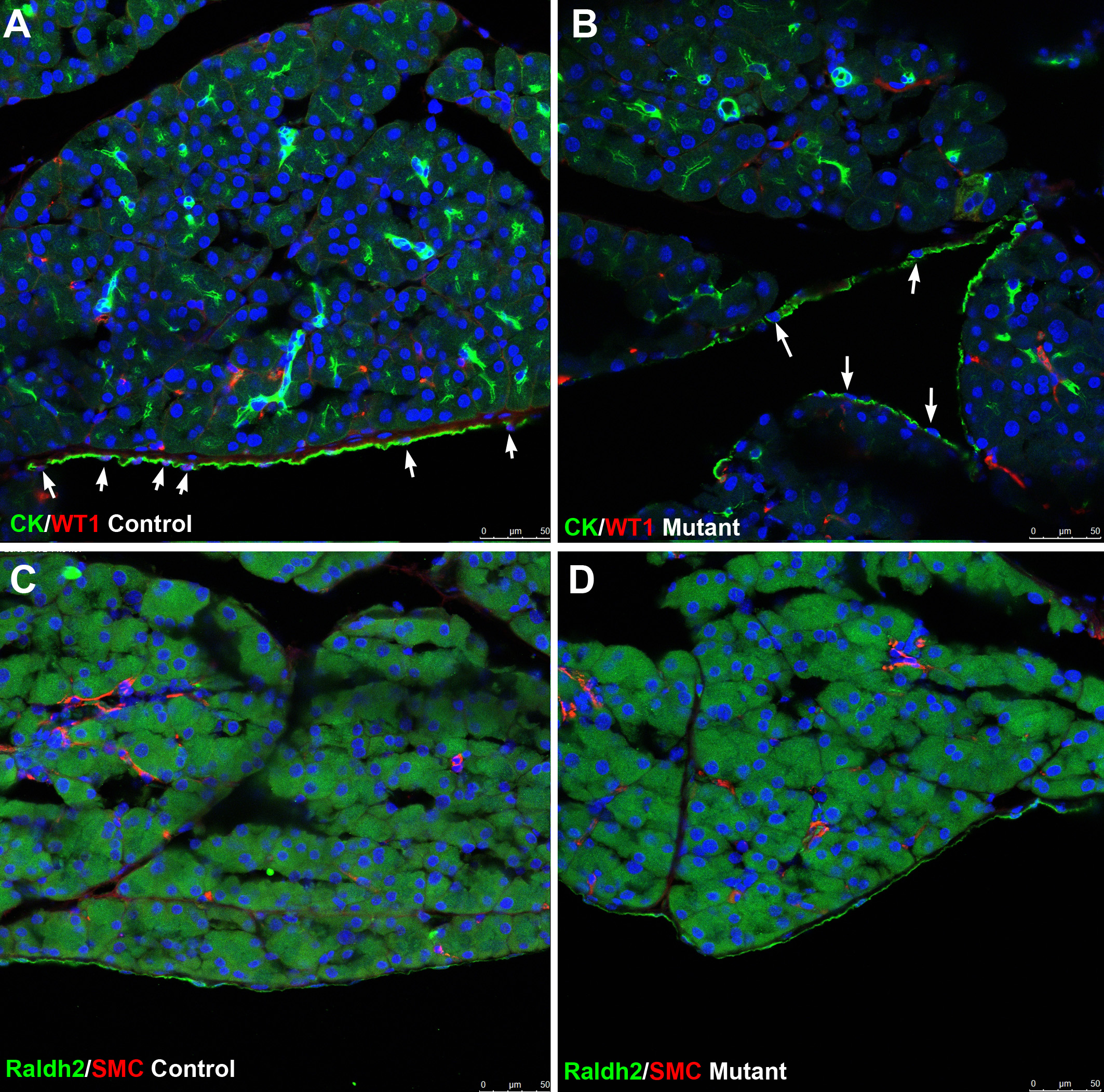

Supplement: S4 Fig — Two doses of tamoxifen are enough for WT1 ablation in the pancreatic mesothelium after five days (arrows in A,B), but this treatment did not activate pancreatic stellate cells, as demonstrated by the lack of upregulation of RALDH2 and SMC α-actin (C,D). Scale bars: 50 μm. (TIF) [file pgen.1007971.s004.tif]

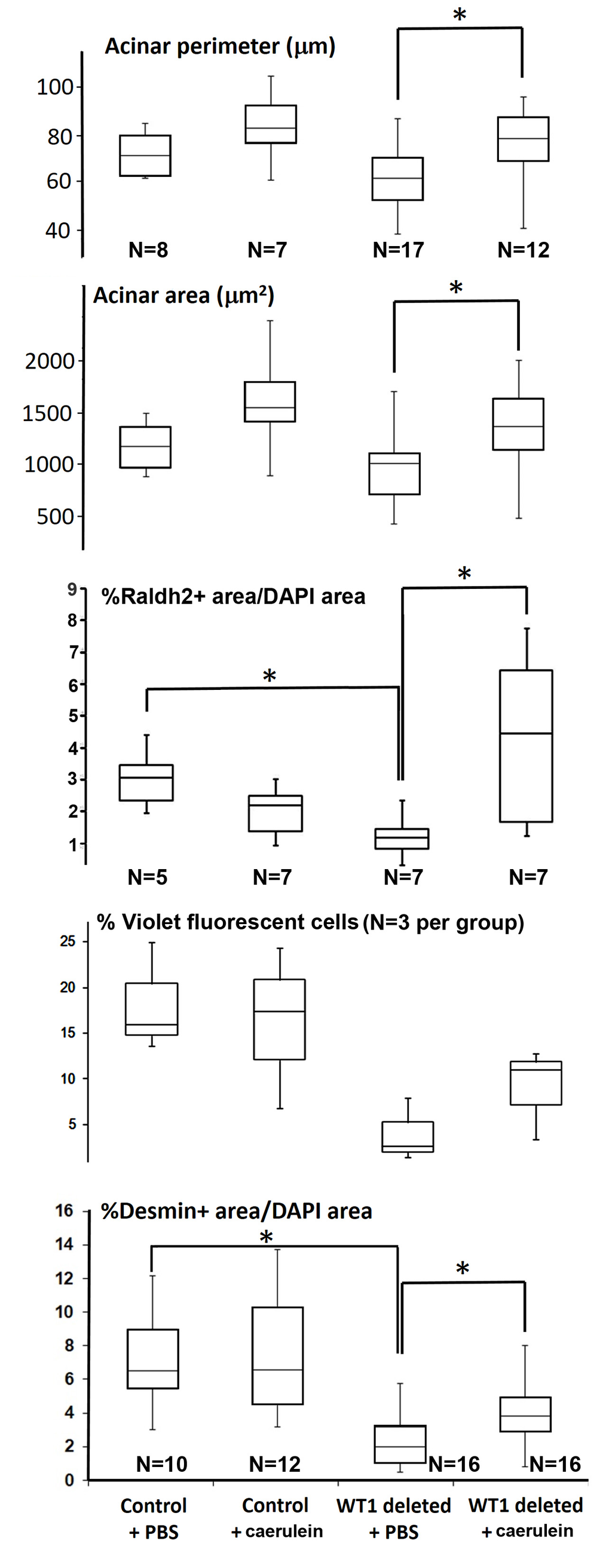

Supplement: S5 Fig — The number of sections analyzed (always obtained from >2 mice per group) is indicated on the figure. The same sections were analyzed for acinar area and perimeter. The percentage of violet-fluorescent cells isolated by centrifugation on Nykodenz solution is also shown. Violet autofluorescence indicates accumulation of retinoids in pancreatic stellate cells. Mean acinar perimeter, mean acinar area, percentage of RALDH2+ and desmin+ area relative to DAPI+ area (nuclei) showed significantly higher values in mice with WT1 deletion after recovery of pancreatitis compared with mice with WT1 deletion (U-Mann Whitney test, p<0.05). The decrease of RALDH2 and desmin immunoreactivity was also significant when comparing control and WT1-deficient mice. Statistical comparison was not applied to the percentages of violet-fluorescent cells because of the low number of biological replicates (N = 3 per group), but the results show the same tendency as the other ones. (TIF) [file pgen.1007971.s005.tif]
