# Supplementary material for: The Wilms’ tumor suppressor gene regulates pancreas homeostasis and repair
Source: PLoS Genet. 2019 Feb 14;15(2):e1007971. doi: 10.1371/journal.pgen.1007971 (PMC6392337; doi:10.1371/journal.pgen.1007971)
Supplement: S1 Table — (PDF) [file pgen.1007971.s006.pdf]

S1 Table. Antibodies used in this study

Antibody	Supplier	Clone or Ref.	Dilution
Monoclonal mouse anti a SMC	Sigma	Clone 1A4-A2547	1/100
Rabbit polyclonal anti-pan cytokeratin	Dako	Z0622	1/200
Rabbit polyclonal anti-laminin	Sigma	L9393	1/200
Rat monoclonal CD105 (endoglin)	eBioscience	14-1051-81	1/100
Rabbit polyclonal anti-ALDH1A2	Abcam	ab75674	1/200
Chicken polyclonal anti-GFP	Abcam	ab 13970	1/200
Mouse monoclonal Anti-E-Cadherin	BD	610181	1/200
Mouse monoclonal anti-Wt1	Millipore	MAB4234	1/100
Rabbit polyclonal anti-GFAP	Dako	Z0334	1/500
Mouse monoclonal anti-Desmin	Sigma	D1033	1/75
Mouse monoclonal Amylase	Santa Cruz	sc-46657	1/200
Hamster Monoclonal anti -Mucin-1	Thermo Fisher	Ab-5	1/200
Rabbit Polyclonal anti-Pan Cadherin	Sigma	E3678	1/100
Mouse Monoclonal anti- PCNA	Sigma	PC10	1/100
Rabbit polyclonal Anti-Caspase 3, Active	Sigma	C8487	1/100
Mouse monoclonal anti-5B5	Abcam	44971	1/100
Mouse monoclonal CD45-PE	eBioscience	12-0451-81	1/100
