# Supplementary material for: The Wilms’ tumor suppressor gene regulates pancreas homeostasis and repair
Source: PLoS Genet. 2019 Feb 14;15(2):e1007971. doi: 10.1371/journal.pgen.1007971 (PMC6392337; doi:10.1371/journal.pgen.1007971)
Supplement: S2 Table — (PDF) [file pgen.1007971.s007.pdf]

S2 Table. Primers used in this study

Gene Target	Forward	Reverse
**Wt1**	gtctttggtgccgtttcagt	gtctttggtgccgtttcagt
**E-Cadherin**	aatggcggcaatgcaatcccaaga	tgccacagaccgattgtggagata
**Snail**	cttgtgtctgcacgacctgt	cttctcaccagtgtgggtgc
**Elastase 1**	aacaacgtggttgcaggctat	cagaacacccagctggacata
**PTF1A**	gcactctctttcctggactga	tccacactttagctgtacgga
**Amylase**	cttgtggcaatgactgggtct	gctgaccattgaccacattcc
**B-actin**	tcctgtggcatccacgaaactaca	accagacagcactgtgttggcata
**Gadph**	tcccattcttccacctttga	ccagggtttcttactccttg
